# Two-Stage Nested Array Direction of Arrival Estimation for Mixed Signals

**DOI:** 10.3390/s22145435

**Published:** 2022-07-21

**Authors:** Wanru Li, Ke Deng

**Affiliations:** Faculty of Electronic and Information Engineering, Xi’an Jiaotong University, Xi’an 710049, China; denke@stu.xjtu.edu.cn

**Keywords:** mixed signals, nested array, sum-difference co-array, oblique projection

## Abstract

In this paper, a novel two-stage subspace-based direction of arrival (DOA) estimation algorithm with the nested array is proposed for mixed signals containing circular and non-circular ones. By exploiting the difference between the two types of steering vectors, the DOAs of circular signals are estimated in the first stage. After eliminating the estimated information of circular signals by the covariance matrix reconstruction and oblique projection methods, the dimensions of the noise subspace are increased in estimating the DOAs of non-circular signals in the second stage. Through the two-stage estimation, the DOAs of the circular and non-circular signals are estimated separately and different types of signals with similar or the same DOAs can be distinguished. Furthermore, to avoid the two-dimensional (2-D) search with huge computational burden, a one-dimensional (1-D) search method exploiting the rank deficiency is proposed in the DOA estimation for non-circular signals. The simulation results show that the proposed algorithm can effectively improve the estimation accuracy and resolution probability, especially when circular and non-circular signals have similar DOAs.

## 1. Introduction

DOA estimation is a key problem in array signal processing and has been widely used in communication, radar, detection, covert transmission, and other fields [1]. A source localization method based on multiple signal classification (MUSIC) was proposed in [2]. The DOAs were calculated by the estimation of signal parameters via the rotational invariant techniques (ESPRIT) in [3], where a novel ESPRIT algorithm based on geometric algebra (GA-ESPRIT) was proposed and the DOA was estimated with double parallel uniform linear arrays (ULA). A fast Fourier transform (FFT)-based method was proposed in [4] with low computational burden. A MUSIC-like method for non-circular signals (NC-MUSIC) was presented in [5], where a virtual array with twice the number of physical array sensors was constructed.

In recent years, the non-uniform linear array (NLA) has been applied to DOA estimation. A compressed sparse array (CSA) was proposed in [6] by combining the compressive sensing and the sparse array. An off-grid DOA estimation method was presented in [7], where a mismatch between true DOA and discrete angular grid was resolved. The estimation performance was improved with NLA and fourth-order cumulants in [8,9]. Specifically, a novel array structure was constructed with the co-prime array in [8], and DOAs was estimated via the sparse Bayesian learning. An array model was proposed in [9], where the fourth-order cumulants and the sparse reconstruction algorithm were exploited to estimate the DOA. An enhanced nested array (ENA) structure was proposed in [10] and a compressed sensing method was applied to DOA estimation. For mixed signals scenarios, the sparse reconstruction and root-MUSIC were used in [11] for non-circular signals and circular signals, respectively. The computational burden of these sparse reconstruction methods is higher than that of the subspace-based ones, although the spatial smoothing is not required.

The methods for co-prime arrays were derived in [12,13], where the DOAs were estimated by exploiting the spatial smoothing and subspace characteristic with the NLA. Specifically, a new filled differential co-prime array (CAFDC) was considered in [12] and the degree of freedom (DOF) was significantly increased by introducing two additional subarrays. The spatial smoothing MUSIC (SS-MUSIC) algorithm was introduced in [14] for circular signals where a ULA with more virtual elements was constructed by the nested array. For 2-D DOA estimation with a coprime cubic array (CCA), a total array-based multiple signals classification (TA-MUSIC) algorithm with a significant increase in accuracy was first proposed in [15]. The traditional nested array was converted into a transformed nested array (TNA) by exchanging the position of the sub-array in [16], and the number of equivalent array elements increased. A method for constructing the continuous differential co-array (cDCA) and continuous sum co-array (cSCA) was proposed for non-circular signals. By discarding the rotation phase of the non-circular signal, the reduced-dimension MUSIC algorithm was proposed, and the computational burden was reduced. To summarize the above description, the sum-difference co-array and reduced-dimension MUSIC (SD-RD-MUSIC) method was illustrated in [17]. To avoid the problem of spatial smoothing with the reduced DOF, a new nested array structure based on quasi-stationary signals (QS) was applied in [18]. The quasi-stationary property of QS was exploited in [19] and the ESPRIT algorithm was applied to estimate the DOAs of non-circular signals. A two-stage DOA estimation algorithm was proposed in [20] with the conformal uniform circular array (UCA), where DOAs were accurately estimated in a multi-target scenario. A spatial-temporal technique was developed in [21] and the low-power spoofing attack and multipath can be distinguished in the global navigation satellite system (GNSS).

In this paper, the scenario of mixed signals containing circular and non-circular ones is considered, and a subspace-based DOA estimation method for two types of signals with the nested array is proposed. The DOAs of the two types of signals are calculated separately based on different types of steering vectors, for the equivalent steering vectors of circular signals only have the cDCA part, while those of non-circular signals contain the cDCA and cSCA parts simultaneously. The DOAs of the circular signals are estimated, while those of non-circular signals are not estimated in the first stage. Then, the covariance matrix containing only the non-circular signals can be obtained by the covariance matrix reconstruction and oblique projection. Thus, the dimensions of noise subspace are increased in estimating the DOAs of non-circular signals in the second stage and two types of signals are distinguished. By separating the DOAs and rotation phases, the DOAs of non-circular signals can be calculated by exploiting the rank deficiency of the matrix, where the 2-D search is avoided and the computational burden is reduced significantly. The estimation accuracy and resolution probability are improved by the separate estimation of different types of signals, especially when circular and non-circular signals have similar DOAs. Even if different types of signals have the same DOAs, the proposed method is still feasible. The methods of estimating different types of signals separately and exploiting the rank deficiency to estimate DOAs in this paper improve the estimation accuracy.

The remainder of this paper is organized as follows. The Section 2 introduces the mixed circular and non-circular signal model, and then explains the structures of the nested array, the cDCA and cSCA. In the Section 3, the method of constructing the cDCA and cSCA using nested array characteristics and estimating DOAs with 1-D search is described. The Section 4 shows the computer simulation results to verify the effectiveness of the proposed method. The Section 5 summarizes the paper.

Notations: We use bold symbols to represent vectors and matrices. The superscripts ${\left(\cdot \right)}^{*}$, ${\left(\cdot \right)}^{T}$, ${\left(\cdot \right)}^{H}$ and ${\left\{\cdot \right\}}^{†}$ denote the conjugation, transpose, conjugate transpose, and pseudo inverse, respectively. E{⋅} represents the calculation of expectation. rank(⋅) denotes the rank of a matrix. diag{⋅} denotes the diagonalization operator. **I** denotes the unit matrix and **O** denotes the matrix with all zero elements.

## 2. Data Model

### 2.1. Mixed Circular and Non-Circular Signals Model

Any digital modulation signal in the complex plane can be expressed as [22]

(1) $$s(t)=\sigma e^{-j\varphi }\left(\sqrt{\frac{\left(1+k\right)}{2}}s_{I}(t)+j\sqrt{\frac{\left(1-k\right)}{2}}s_{Q}(t)\right)$$

where φ denotes the rotation phase, *k* controls the signal amplitude, $s_{I}(t)$ denotes the co-directional component, and $s_{Q}(t)$ denotes the orthogonal component. $E\{|s_{I}(t){|}^{2}\}=1$, $E\{|s_{Q}(t){|}^{2}\}=1$ and $E\{s_{I}(t)s_{Q}(t)\}=0$.

To measure the degree of non-circularity, the definition of a signal’s non-circular rate ρ is given by [23]

(2) $$\rho =\frac{E\{s^{2}(t)\}}{E\{|s(t){|}^{2}\}}=ke^{j2\varphi }\;0\leq |\rho |\leq 1$$

where |ρ|=0 and 0<|ρ|≤1 represent the circular and non-circular signals, respectively, and |ρ|=1 indicates the strictly non-circular signal. For convenience, only the case of mixed strictly non-circular and circular signals is considered in this paper. Then the circular signal can be represented as $s_{c}(t)=e^{-j\varphi }s_{IQ}(t)$, and the non-circular signal can be represented as $s_{n}(t)=e^{-j\varphi }s_{II}(t)$, where $s_{IQ}(t)=\sigma \left(\sqrt{\frac{1}{2}}s_{I}(t)+j\sqrt{\frac{1}{2}}s_{Q}(t)\right)$ and $s_{II}(t)=\sigma s_{I}(t)$.

For the rest of this paper, the assumptions are required as follows:

(1) All signals are statistically independent, zero-mean uncorrelated narrowband stationary processes.
(2) Every sensor has zero-mean, additive white Gaussian noise and the noise is independent from the signals.

### 2.2. The Nested Array, cDCA, and cSCA Model

Consider a nested array having $N_{1}$ elements with inter-element spacing d and $N_{2}$ elements with inter-element spacing $\left(N_{1}+1\right)d$, where d is $\frac{\lambda }{2}$ in this paper and λ denotes the wavelength of the carrier wave. Assume L to be a set of integers, defined as

(3) $$L=\left\{n_{1}|0\leq n_{1}\leq N_{1}-1\right\}\cup \left\{n_{2}(N_{1}+1)-1|1\leq n_{2}\leq N_{2}\right\}$$

Then the sensor locations of the nested array can be described as

(4) $$L^{\prime }=dL=\left\{l_{1},l_{2},\cdots ,l_{N^{\prime }}\right\}$$

where $N^{\prime }=N_{1}+N_{2}$ is the total number of array elements.

**Figure 1:**
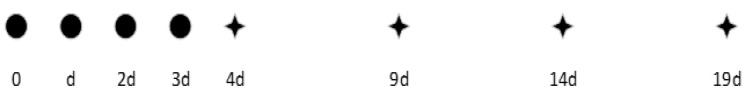
Nested array model.

Figure 1 shows the model of the nested array, where $N_{1}=N_{2}=4$.

Assume *M* uncorrelated narrowband signals impinge on a nested array. Then, the received signals on the array can be expressed as

(5) $$x(t)=s(t)+n(t)=A_{C}s_{C}(t)+A_{N}s_{N}(t)+n(t)=x_{C}(t)+x_{N}(t)+n(t)$$

where **x**(*t*) and **n**(*t*) are $N^{\prime }\times 1$ vectors, and

(6) $$x_{C}(t)=A_{C}s_{C}(t)$$

(7) $$x_{N}(t)=A_{N}s_{N}(t)$$

(8) $$s(t)=A_{C}s_{C}(t)+A_{N}s_{N}(t)$$

(9) $$A_{N}=[a(\theta_{1}),a(\theta_{2}),\cdots ,a(\theta_{M_{1}})]$$

(10) $$A_{C}=[a(\theta_{M_{1}+1}),a(\theta_{M_{1}+2}),\cdots ,a(\theta_{M})]$$

(11) $$s_{N}(t)={\left[s_{1}(t),s_{2}(t),\cdots ,s_{M_{1}}(t)\right]}^{T}$$

(12) $$s_{C}(t)={\left[s_{M_{1}+1}(t),s_{M_{1}+2}(t),\cdots ,s_{M}(t)\right]}^{T}$$

$a(\theta_{m})={\left[e^{-j2\pi l_{1}sin\theta_{m}/\lambda },e^{-j2\pi l_{2}sin\theta_{m}/\lambda },\ldots ,\;e^{-j2\pi l_{N^{\prime }}sin\theta_{m}/\lambda }\right]}^{T}$ denotes the *m*-th steering vector and $M_{1}$ indicates the number of non-circular signals.

**Figure 2:**
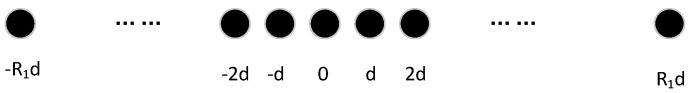
cDCA model.

**Figure 3:**
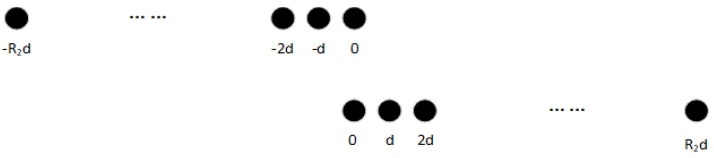
Negative and positive cSCA model.

Figure 2 and Figure 3 show the locations and number of elements in the cDCA and cSCA, respectively. According to [17], the total number of elements in the cDCA is $2R_{1}+1$, and the number of elements in the cSCA is $2(R_{2}+1)$, where $R_{1}=N_{1}N_{2}+N_{2}-1$ and $R_{2}=N_{1}N_{2}+N_{1}+N_{2}-1$. The distance between the adjacent virtual elements is *d*.

## 3. The Proposed Method

Define the matrix **J** as

(13) $$J=\left(\begin{array}{l} x(t)\\ x^{*}(t) \end{array}\right)$$

Then, the covariance matrix of **J** can be expressed as

(14) $$R_{J}=E\{JJ^{H}\}=\left(\begin{array}{cc} E\{x(t)x^{H}(t)\} & E\{x(t)x^{T}(t)\}\\ E\{x^{*}(t)x^{H}(t)\} & E\{x^{*}(t)x^{T}(t)\} \end{array}\right)$$

For convenience, $R_{J}$ is further rewritten as

(15) $$R_{J}=R_{C}+R_{N}+\sigma_{n}^{2}I$$

where

(16) $$R_{C}=\left(\begin{array}{cc} E\{x_{C}(t)x_{C}^{H}(t)\} & E\{x_{C}(t)x_{C}^{T}(t)\}\\ E\{x_{C}^{*}(t)x_{C}^{H}(t)\} & E\{x_{C}^{*}(t)x_{C}^{T}(t)\} \end{array}\right)$$

and

(17) $$R_{N}=\left(\begin{array}{cc} E\{x_{N}(t)x_{N}^{H}(t)\} & E\{x_{N}(t)x_{N}^{T}(t)\}\\ E\{x_{N}^{*}(t)x_{N}^{H}(t)\} & E\{x_{N}^{*}(t)x_{N}^{T}(t)\} \end{array}\right)$$

$\sigma_{n}^{2}$ represents the noise power.

### 3.1. Non-Circular Signals

For non-circular signals, only $E\{x_{N}(t)x_{N}^{H}(t)\}$, $E\{x_{N}(t)x_{N}^{T}(t)\}$, and $E\{x_{N}^{*}(t)x_{N}^{H}(t)\}$ are analyzed because all valid information of $E\{x_{N}^{*}(t)x_{N}^{T}(t)\}$ is also contained in $E\{x_{N}(t)x_{N}^{H}(t)\}$.

(18) $$E\{x_{N}(t)x_{N}^{H}(t)\}=A_{N}diag\{\sigma_{1}^{2},\sigma_{2}^{2},\cdots ,\sigma_{M_{1}}^{2}\}A_{N}^{H}=\sum_{m=1}^{M_{1}}\sigma_{m}^{2}a(\theta_{m})a{\left(\theta_{m}\right)}^{H}$$

(19) $$E\{x_{N}(t)x_{N}^{T}(t)\}=A_{N}\Phi_{N}diag\{\sigma_{1}^{2},\sigma_{2}^{2},\cdots ,\sigma_{M_{1}}^{2}\}\Phi_{N}^{T}A_{N}^{T}$$

where $\sigma_{m}^{2}$ represents the power of the *m*-th non-circular signal and $\Phi_{N}=diag\{e^{-j\varphi_{1}},e^{-j\varphi_{2}},\cdots ,e^{-j\varphi_{M_{1}}}\}$ is a diagonal matrix which contains the rotation phases information of the non-circular signals. We define the merged direction matrix $A_{v}$ and its *m*-th steering vector as

(20) $$A_{v}=A_{N}\Phi_{N}=[a_{v}(\theta_{1},\varphi_{1}),a_{v}(\theta_{2},\varphi_{2}),\cdots ,a_{v}(\theta_{M_{1}},\varphi_{M_{1}})]$$

(21) $$a_{v}(\theta_{m},\varphi_{m})=a_{N}(\theta_{m})e^{-j\varphi_{m}}$$

Then $E\{x_{N}(t)x_{N}^{T}(t)\}$ can be rewritten as

(22) $$\begin{array}{ll} E\{x_{N}(t)x_{N}^{T}(t)\} & =A_{v}diag\{\sigma_{1}^{2},\sigma_{2}^{2},\cdots ,\sigma_{M_{1}}^{2}\}A_{v}^{T}\\ & =\sum_{m=1}^{M_{1}}\sigma_{m}^{2}a_{v}(\theta_{m},\varphi_{m})a_{v}{\left(\theta_{m},\varphi_{m}\right)}^{T} \end{array}$$

And

(23) $$E\{x_{N}^{*}(t)x_{N}^{H}(t)\}={\left(E\{x_{N}(t)x_{N}^{T}(t)\}\right)}^{*}$$

According to [17], after vectorizing and removing the redundant elements in (18), (22) and (23), the equivalent signals on the cDCA and cSCA can be expressed as

(24) $$y_{v}=D_{n}p_{n}$$

(25) $$D_{n}=[d_{n}(\theta_{1}),d_{n}(\theta_{2}),\cdots ,d_{n}(\theta_{M_{1}})]$$

(26) $$y_{\delta }=C_{\delta }p_{n}$$

(27) $$y_{\phi }=C_{\phi }p_{n}$$

(28) $$C_{\delta }=[c_{\delta }(\theta_{1},\varphi_{1}),c_{\delta }(\theta_{2},\varphi_{2}),\cdots ,c_{\delta }(\theta_{M_{1}},\varphi_{M_{1}})]$$

(29) $$C_{\phi }=[c_{\phi }(\theta_{1},\varphi_{1}),c_{\phi }(\theta_{2},\varphi_{2}),\cdots ,c_{\phi }(\theta_{M_{1}},\varphi_{M_{1}})]$$

### 3.2. Circular Signals

According to the definition of circular signals, we have $E\{x_{C}(t)x_{C}^{T}(t)\}=O$ and $E\{x_{C}^{*}(t)x_{C}^{H}(t)\}=O$ in (16). Then, the information of circular signals only exists in $E\{x_{C}(t)x_{C}^{H}(t)\}$ and the cDCA parts of the steering vectors of circular signals have the same structure as those of non-circular signals. The circular signals on the cDCA are

(30) $$y_{c}=D_{c}p_{c}$$

where $D_{c}=[d_{c}(\theta_{M_{1}+1}),d_{c}(\theta_{M_{1}+2}),\cdots ,d_{c}(\theta_{M})]$ and $p_{c}={\left[\sigma_{M_{1}+1}^{2},\sigma_{M_{1}+2}^{2},\cdots ,\sigma_{M}^{2}\right]}^{T}$ represents the power vector of the circular signals.

### 3.3. DOA Estimation of Mixed Signals

According to the noise assumption, noise only exists on the cDCA. Then, the constructed signals and noise on the cDCA can be expressed as

(31) $$y_{d}=D_{d}p+\sigma_{n}^{2}n_{d}$$

(32) $$D_{d}=[d_{n}(\theta_{1}),\cdots ,d_{n}(\theta_{M_{1}}),d_{c}(\theta_{M_{1}+1}),\cdots ,d_{c}(\theta_{M})]$$

(33) $$p={\left[\sigma_{1}^{2},\cdots ,\sigma_{M_{1}}^{2},\sigma_{M_{1}+1}^{2},\cdots \sigma_{M}^{2}\right]}^{T}$$

$n_{d}$ is a column vector in which only the $R_{1}+1$ -th element is 1 and the other elements are 0.

The constructed signals on the cSCA can be expressed as

(34) $$y_{\delta }=S_{z}p$$

(35) $$y_{\phi }=S_{f}p$$

(36) $$S_{z}=[c_{\delta }(\theta_{1},\varphi_{1}),\cdots ,c_{\delta }(\theta_{M_{1}},\varphi_{M_{1}}),\nu_{M_{1}+1},\cdots ,\nu_{M}]$$

(37) $$S_{f}=[c_{\phi }(\theta_{1},\varphi_{1}),\cdots ,c_{\phi }(\theta_{M_{1}},\varphi_{M_{1}}),\tau_{M_{1}+1},\cdots ,\tau_{M}]$$

where $\nu_{m}\;(M_{1}+1\leq m\leq M)$ and $\tau_{m}\;(M_{1}+1\leq m\leq M)$ are all zero vectors.

So far, the structures of the cDCA and cSCA have been derived. The equivalent received signal vector ***p*** can be regarded as the signals of a single snapshot, which needs to be smoothed to increase the rank of the covariance matrix in our method. Here, the method of smoothing the signals on the cDCA, the positive and negative cSCA separately is just like that in [17].

First, the signals on the cDCA are smoothed. In the cDCA, the steering vectors of circular signals and non-circular signals have the same form, then $D_{e}$ can be expressed as

(38) $$D_{e}=[d_{e}(\theta_{1}),d_{e}(\theta_{2}),\cdots ,d_{e}(\theta_{M})]$$

the *m*-th steering vector is

(39) $$d_{e}(\theta_{m})={\left[1,e^{-j\pi \sin\theta_{m}},e^{-j2\pi \sin\theta_{m}},\cdots ,e^{-jR_{1}\pi \sin\theta_{m}}\right]}^{T}$$

And

(40) $$\begin{array}{ll} Y_{e} & =D_{e}[p,Gp,\ldots ,G^{R_{1}}p]+\sigma_{n}^{2}I_{R_{1}+1}\\ & =D_{e}S_{d}+\sigma_{n}^{2}I_{R_{1}+1} \end{array}$$

where

(41) $$G=\left(\begin{array}{cccc} e^{j\pi \sin\theta_{1}} & & & \\ & e^{j\pi \sin\theta_{2}} & & \\ & & \ddots & \\ & & & e^{j\pi \sin\theta_{M}} \end{array}\right)$$

Next, the signals on the positive and negative cSCA are smoothed separately. To ensure that the smoothed matrix has the same number of columns as $Y_{e}$, the positive and negative cSCA are also divided into $R_{1}+1$ subarrays, and each subarray contains $R_{2}-R_{1}+1$ elements. Then, the constructed equivalent signals can be expressed as

(42) $$y_{zs_{i}}=S_{\delta }G^{i-1}p$$

(43) $$y_{fs_{i}}=S_{\phi }G^{i-1}p$$

where

(44) $$S_{\delta }=[\varpi_{1},\cdots ,\varpi_{M_{1}},\varpi_{M_{1}+1},\cdots ,\varpi_{M}]$$

(45) $$S_{\phi }=[\varsigma_{1},\cdots ,\varsigma_{M_{1}},\varsigma_{M_{1}+1},\cdots ,\varsigma_{M}]$$

(46) $$\varpi_{m}={\left[e^{-jR_{1}\pi \sin\theta_{m}}e^{-j2\varphi_{m}},e^{-j(R_{1}+1)\pi \sin\theta_{m}}e^{-j2\varphi_{m}},\cdots ,e^{-jR_{2}\pi \sin\theta_{m}}e^{-j2\varphi_{m}}\right]}^{T}\;1\leq m\leq M_{1}$$

(47) $$\varpi_{m}=O_{(R_{2}-R_{1}+1)\times 1}\;M_{1}+1\leq m\leq M$$

(48) $$\varsigma_{m}={\left[e^{j(R_{2}-R_{1})\pi \sin\theta_{m}}e^{j2\varphi_{m}},\cdots ,e^{j\pi \sin\theta_{m}}e^{j2\varphi_{m}},e^{j2\varphi_{m}}\right]}^{T}\;1\leq m\leq M_{1}$$

(49) $$\varsigma_{m}=O_{(R_{2}-R_{1}+1)\times 1}\;M_{1}+1\leq m\leq M$$

By arranging the equivalent signals on the $R_{1}+1$ subarrays, the spatially smoothed signal matrices $Y_{\beta }$ and $Y_{\sigma }$ can be rewritten as

(50) $$\begin{array}{l} Y_{\beta }=[y_{zs_{1}},y_{zs_{2}},\ldots ,y_{zs_{R_{1}+1}}]\\ \;\;\;\;\;=S_{\delta }[p,Gp,\cdots ,G^{R_{1}}p]=S_{\delta }S_{d} \end{array}$$

(51) $$\begin{array}{l} Y_{\sigma }=[y_{fs_{1}},y_{fs_{2}},\cdots ,y_{fs_{R_{1}+1}}]\\ \;\;\;\;\;=S_{\phi }[p,Gp,\cdots ,G^{R_{1}}p]=S_{\phi }S_{d} \end{array}$$

$Y_{\beta }$, $Y_{e}$, and $Y_{\sigma }$ are concatenated as (52) to form a new received signal matrix **Y**.

(52) $$Y=\left[\begin{array}{l} Y_{\sigma }\\ Y_{e}\\ Y_{\beta } \end{array}\right]=\left[\begin{array}{l} S_{\phi }\\ D_{e}\\ S_{\delta } \end{array}\right]S_{d}+\left[\begin{array}{l} O_{\left(R_{2}-R_{1}+1)\times (R_{1}+1\right)}\\ \;\;\;\;\;\sigma_{n}^{2}I_{R_{1}+1}\\ O_{\left(R_{2}-R_{1}+1)\times (R_{1}+1\right)} \end{array}\right]$$

For convenience, we define $A_{s}=\left[\begin{array}{l} S_{\phi }\\ D_{e}\\ S_{\delta } \end{array}\right]$ and $N_{q}=\left[\begin{array}{l} O_{\left(R_{2}-R_{1}+1)\times (R_{1}+1\right)}\\ \;\;\;\;\;\sigma_{n}^{2}I_{R_{1}+1}\\ O_{\left(R_{2}-R_{1}+1)\times (R_{1}+1\right)} \end{array}\right]$

(53) $$Y=A_{s}S_{d}+N_{q}$$

where $A_{s}=[a_{s_{1}},\cdots ,a_{s_{M_{1}}},a_{s_{M_{1}+1}}\cdots ,a_{s_{M}}]$

(54) $$a_{s_{m}}=\left[\begin{array}{l} \varsigma_{m}(\theta_{m},\varphi_{m})\\ \;d_{e}(\theta_{m})\\ \varpi_{m}(\theta_{m},\varphi_{m}) \end{array}\right]\;1\leq m\leq M_{1}$$

(55) $$a_{s_{m}}=\left[\begin{array}{l} O_{(R_{2}-R_{1}+1)\times 1}\\ \;d_{e}(\theta_{m})\\ O_{(R_{2}-R_{1}+1)\times 1} \end{array}\right]\;\;\;\;M_{1}+1\leq m\leq M$$

After these steps, an uneven nested array can be transformed into a ULA, and the number of equivalent elements is significantly increased.

The covariance matrix of **Y** can be calculated as

(56) $$R_{\chi }=\frac{1}{R_{1}+1}YY^{H}$$

The eigenvalues decomposition of $R_{\chi }$ can be expressed as

(57) $$R_{\chi }=U_{sc}\Lambda_{sc}U_{sc}^{H}+U_{nc}\Lambda_{nc}U_{nc}^{H}$$

$\Lambda_{nc}=diag\{\lambda_{M+1},\lambda_{M+2},\cdots \lambda_{2R_{2}-R_{1}+3}\}$ is a diagonal matrix in which the diagonal elements are the $2R_{2}-R_{1}+3-M$ small eigenvalues, and their corresponding eigenvector matrix $U_{nc}$ constitutes the noise subspace.

It can be seen from (54) and (55) that the cSCA parts of the steering vectors of the circular signals are zero vectors while those of the non-circular signals contain the angles and rotation phases information. By exploiting the difference between two types of steering vectors, we can distinguish the two types of signals and estimate their DOAs separately.

#### 3.3.1. DOAs of Circular Signals

When $M_{1}+1\leq m\leq M$, $a_{s_{m}}=\left[\begin{array}{l} O_{(R_{2}-R_{1}+1)\times 1}\\ \;d_{e}(\theta_{m})\\ O_{(R_{2}-R_{1}+1)\times 1} \end{array}\right]$. By exploiting the orthogonal property of the signal subspace and noise subspace, the space spectrum $P_{1}$ can be expressed as

(58) $$P_{1}(\theta )=\frac{1}{a_{s_{m}}^{H}(\theta )U_{nc}U_{nc}^{H}a_{s_{m}}(\theta )}\;$$

After the one-dimensional spectral peak search, the angles corresponding to the maximum $M-M_{1}$ peaks are the DOAs of circular signals. Due to the differences in steering vectors of different types of signals, only the DOAs of circular signals can be estimated in the peak search of (58). Since the cDCA part is contained in the steering vectors of both non-circular and circular signals, the information of circular signals on the cDCA needs to be eliminated after the DOAs of circular signals are estimated.

#### 3.3.2. Eliminate the Information of Circular Signals

Two methods are proposed to eliminate the estimated circular signals based on the total number of signals.

- Reconstruct the covariance matrix

When the total number of circular and non-circular signals does not exceed $N^{\prime }$, the power of the circular signals can be calculated from the covariance matrix $E\{x(t)x^{H}(t)\}$, and the information of circular signals can be eliminated like the method in [24].

From the previous derivation, the information of the circular signals only exists in $E\{x(t)x^{H}(t)\}$. Let $R_{h}$ be the covariance matrix without noise. From the DOAs estimation of circular signals, the power can be calculated as

(59) $$\sigma_{m}^{2}={\{a^{H}(\theta_{m})R_{h}^{†}a(\theta_{m})\}}^{-1}\;\;\;M_{1}+1\leq m\leq M$$

Then the covariance matrix $E\{x_{N}(t)x_{N}^{H}(t)\}$ containing only DOAs of non-circular signals is obtained as

(60) $$E\{x_{N}(t)x_{N}^{H}(t)\}=R_{h}-A_{C}(\theta )diag\{\sigma_{M_{1}+1}^{2},\sigma_{M_{1}+2}^{2},\cdots ,\sigma_{M}^{2}\}A_{C}^{H}(\theta )$$

Then, the redundant elements in $E\{x_{N}(t)x_{N}^{H}(t)\}$ are removed. According to the smoothing method in (38)–(41), we have

(61) $$Y_{\varepsilon }=[y_{n_{1}},y_{n_{2}},\cdots ,y_{n_{R_{1}+1}}]=D_{\varepsilon }[p_{n},G_{1}p_{n},\ldots ,G_{1}^{R_{1}}p_{n}]=D_{\varepsilon }S_{n}$$

(62) $$G_{1}=\left(\begin{array}{cccc} e^{j\pi \sin\theta_{1}} & & & \\ & e^{j\pi \sin\theta_{2}} & & \\ & & \ddots & \\ & & & e^{j\pi \sin\theta_{M_{1}}} \end{array}\right)$$

(63) $$D_{\varepsilon }=[\varepsilon (\theta_{1}),\varepsilon (\theta_{2}),\cdots ,\varepsilon (\theta_{M_{1}})]$$

$p_{n}={\left[\sigma_{1}^{2},\sigma_{2}^{2},\cdots ,\sigma_{M_{1}}^{2}\right]}^{T}$ represents the power vector of the non-circular signals.

The equivalent signals on the cSCA do not contain the information of circular signals and noise, therefore, it is not necessary to smooth the signals on the cSCA again when calculating the DOAs of non-circular signals.

By splicing $Y_{\sigma }$, $Y_{\varepsilon }$ and $Y_{\beta }$ together, we can obtain the equivalent received non-circular signals matrix after removing noise and circular signals information.

(64) $$Y_{n}=\left[\begin{array}{l} Y_{\sigma }\\ Y_{\varepsilon }\\ Y_{\beta } \end{array}\right]=A_{\varepsilon }S_{n}$$

where $A_{\varepsilon }=[a_{\varepsilon }(\theta_{1},\varphi_{1}),a_{\varepsilon }(\theta_{2},\varphi_{2})\cdots ,a_{\varepsilon }(\theta_{M_{1}},\varphi_{M_{1}})]$ and $a_{\varepsilon }(\theta_{m},\varphi_{m})=\left[\begin{array}{l} \varsigma_{m}(\theta_{m},\varphi_{m})\\ \;\;\;\;\varepsilon (\theta_{m})\\ \varpi_{m}(\theta_{m},\varphi_{m}) \end{array}\right]$

The covariance matrix of the non-circular signals can be calculated as

(65) $$R_{\nu }=\frac{1}{R_{1}+1}Y_{n}Y_{n}^{H}$$

- Oblique projection

The above method is no longer applicable when the number of signals exceeds $N^{\prime }$. Another method is proposed by exploiting the oblique projection method. The proof of the oblique projection method is presented in the Appendix A. The oblique projection operator is constructed as

(66) $$E_{A_{SC}(\theta )A_{SN}}=A_{SC}(\theta ){\left(A_{SC}^{H}(\theta )R_{\chi }^{†}A_{SC}(\theta )\right)}^{-1}A_{SC}^{H}(\theta )R_{\chi }^{†}$$

where $A_{SC}(\theta )$ donates the equivalent steering vector matrix of the smoothed circular signals in (53). Then, the covariance matrix is obtained as

(67) $$R_{\nu }=(I-E_{A_{SC}(\theta )A_{SN}})R_{\chi }{\left(I-E_{A_{SC}(\theta )A_{SN}}\right)}^{H}$$

It should be noted that the oblique projection algorithm is also applicable when the total number of signals is no more than $N^{\prime }$.

#### 3.3.3. DOAs of Non-Circular Signals

Through the above derivation, the two methods for eliminating circular signals are proposed, and the covariance matrix $R_{\nu }$ of non-circular signals is obtained. By exploiting the eigenvalue decomposition, $R_{\nu }$ can be expressed as

(68) $$R_{\nu }=U_{sn}\Lambda_{sn}U_{sn}^{H}+U_{nn}\Lambda_{nn}U_{nn}^{H}$$

The eigenvectors corresponding to the smaller $2R_{2}-R_{1}+3-M_{1}$ eigenvalues represent the noise subspace. According to the subspace theory, the signal subspace and noise subspace are orthogonal.

(69) $$P_{2}(\theta ,\varphi )=\frac{1}{a_{\varepsilon }^{H}(\theta ,\varphi )U_{nn}U_{nn}^{H}a_{\varepsilon }(\theta ,\varphi )}$$

After performing a 2-D spectral peak search on (69), the DOAs and rotation phases of non-circular signals can be obtained. To reduce the amount of calculation, a method converting the 2-D search into 1-D search is proposed.

$a_{\varepsilon }(\theta_{m},\varphi_{m})$ is rewritten as the form of the multiplication of two matrices in (70).

(70) $$a_{\varepsilon }(\theta_{m},\varphi_{m})=[\begin{array}{c} e^{j(R_{2}-R_{1})\pi \sin\theta_{m}}e^{i2\varphi_{m}}\\ \vdots \\ e^{j\pi \sin\theta_{m}}e^{j2\varphi_{m}}\\ e^{j2\varphi_{m}}\\ 1\\ e^{-j\pi \sin\theta_{m}}\\ \vdots \\ e^{-jR_{1}\pi \sin\theta_{m}}\\ e^{-jR_{1}\pi \sin\theta_{m}}e^{-j2\varphi_{m}}\\ e^{-j(R_{1}+1)\pi \sin\theta_{m}}e^{-j2\varphi_{m}}\\ \vdots \\ e^{-jR_{2}\pi \sin\theta_{m}}e^{-j2\varphi_{m}} \end{array}]=\underset{Q}{\underbrace{\left[\begin{array}{ccc} e^{j(R_{2}-R_{1})\pi \sin\theta_{m}} & & \\ \vdots & & \\ e^{j\pi \sin\theta_{m}} & & \\ 1 & & \\ & 1 & \\ & e^{-j\pi \sin\theta_{m}} & \\ & \vdots & \\ & e^{-jR_{1}\pi \sin\theta_{m}} & \\ & & e^{-jR_{1}\pi \sin\theta_{m}}\\ & & e^{-j(R_{1}+1)\pi \sin\theta_{m}}\\ & & \vdots \\ & & e^{-jR_{2}\pi \sin\theta_{m}} \end{array}\right]}}\left[\begin{array}{c} e^{j2\varphi_{m}}\\ 1\\ e^{-j2\varphi_{m}} \end{array}\right]$$

It can be seen from (70) that **Q** only contains the angle information of the signals, and $\Xi =\left[\begin{array}{l} \chi_{1}\\ \chi_{2}\\ \chi_{3} \end{array}\right]=\left[\begin{array}{c} e^{j2\varphi_{m}}\\ 1\\ e^{-j2\varphi_{m}} \end{array}\right]$ only contains the rotation phase information. From the subspace theory, when both the angle and phase are correct

(71) $$U_{nn}^{H}a_{\varepsilon }(\theta_{m},\varphi_{m})=O_{(2R_{2}-R_{1}+3-M_{1})\times 1}\;1\leq m\leq M_{1}$$

Let

(72) $$K=U_{nn}^{H}Q$$

**K** is considered to be a coefficient matrix and Ξ is the solution vector. Here, the problem can be transformed into the homogeneous equation KΞ=O existing at least one non-zero solution $\Xi =\left[\begin{array}{c} e^{j2\varphi_{m}}\\ 1\\ e^{-j2\varphi_{m}} \end{array}\right]$, which illustrates that the rank of the coefficient matrix **K** is less than the number of unknowns. Therefore, the rank estimator method can be used to estimate the DOAs of non-circular signals.

(73) rank(K)<3

Since all DOAs are in $\left[-90^{\circ },90^{\circ }\right]$, they can be obtained by searching θ. Because the rank of **K** changes with different θ, the correct DOAs can obtained when rank of **K** is deficient. The rotational phases can be further estimated from the singular vectors of **K** in our method, but they are not considered in many papers [5,14,17] and also in our simulations.

If the traditional 2-D search is applied to (69), it can be summarized as:

$$\;\mathrm{The search range of}\;\theta \;\mathrm{is}[-90^{\circ },90^{\circ }]\;\;\mathrm{The search range of}\;\Phi \;\mathrm{is}[-180^{\circ },180^{\circ }]\;\;\mathrm{FOR}\;\theta =-90^{\circ },-90^{\circ }+\Delta \theta ,\;\cdots {\;90}^{\circ }\;\;\mathrm{FOR}\;\phi =-180^{\circ },-180^{\circ }+\Delta \phi ,\;\cdots \;180^{\circ }\;\;P_{2}(\theta ,\varphi )=\frac{1}{a_{\varepsilon }^{H}(\theta ,\varphi )U_{nn}U_{nn}^{H}a_{\varepsilon }(\theta ,\varphi )}$$

where Δϕ and Δθ denote the search interval of the rotation phase and DOA, respectively.

The proposed 1-D search method in this paper avoids the 2-D search and the computational burden is significantly reduced. The angle search process of the proposed method is summarized as:

FOR θ=−90∘,−90∘+Δθ, ⋯ 90∘Construct Q in (70)K=UnnHQCalculate the rank of K

It can be seen that the proposed method only needs to perform 1-D search for one time in estimating DOAs of non-circular signals and the computational burden is low.

In summary, the main steps of the proposed method can be described as follows:

(1) Construct matrix **J** via (13) and calculate covariance matrix $R_{J}$ via (14).
(2) Construct $y_{d}$, $y_{\delta }$, and $y_{\phi }$ from the covariance matrix and exploit spatial smoothing.
(3) The equivalent sum-difference co-array steering vectors are constructed by (52), and the DOAs of circular signals are determined by the spectral peak search in (58).
(4) When the total number of signals does not exceed the actual number of elements $N^{\prime }$, use the method in step i. Otherwise, use the method in step ii.
  - The covariance matrix of the reconstructed circular signals is eliminated by (60). After the related elements being extracted and smoothed, the covariance matrix of non-circular signals can be obtained as (65).
  - Calculate the oblique projection operator based on the estimated DOAs of the circular signals in (66). Then, the covariance matrix of the non-circular signals can be obtained by (67).
(5) Perform eigenvalue decomposition on the covariance matrix to obtain the noise subspace. The steering vector of the non-circular signal is decomposed as (70). Then, construct matrix **K** in (72) and the DOAs of non-circular signals are determined by searching for the rank deficiency of **K**.

##### Discussion A: Computational Complexity

For convenience, we define Δθ as the angle search interval and F as the number of virtual array elements. $M_{2}=M-M_{1}$ is the number of circular signals. The computational complexity of [17] mainly comes from calculating the inverse of the matrix for both circular and non-circular signals. The overall complexity of this method is $O\left(F^{3}+F^{2}(R_{1}+1)+\frac{\pi }{\Delta \theta }(6F(F-M)+9F+21)\right)$. The complexity of the proposed algorithm mainly comes from the removal of circular signals and the singular value decomposition. If the covariance matrix reconstruction method is used, the complexity is $O\left(2F^{3}+N^{\prime 3}+M_{2}(N^{\prime }+2N^{\prime 2})+N^{\prime }M_{2}^{2}+2F^{2}(R_{1}+1)+\frac{\pi }{\Delta \theta }(5F^{2}-2FM-3FM_{1}-9M_{1}+10F+9)\right)$. If the oblique projection method is used, the complexity is $O\left(6F^{3}+M_{2}^{3}+2FM_{2}^{2}+F^{2}(2M_{2}+R_{1}+1)+\frac{\pi }{\Delta \theta }(5F^{2}-2FM-3FM_{1}-9M_{1}+10F+9)\right)$.

By estimating different types of signals separately, the complexity is increased, but the accuracy of estimation is also improved. When the different types of signals are estimated simultaneously in our method, the complexity mainly comes from singular value decomposition, which is similar to the complexity in [17].

##### Discussion B: The Scenario of Unmixed Signals

The proposed method can also work well for unmixed signals. For circular signals, only cDCA part and one parameter θ exist in this problem, and the traditional MUSIC algorithm can be exploited to estimate the DOA directly through the 1-D spectral peak search. For non-circular signals, the problem in (70) can be transformed into the homogeneous equation existing at least one non-zero solution and the rank of the coefficient matrix **K** is less than three, which has been discussed in Section 3.3.3. To sum up, the proposed method can be applied to the scenario of unmixed signals, and only the one-stage estimation is needed.

## 4. Simulation Results

In this section, simulation results are presented to verify the performance of our algorithm where the condition number method is chosen as the indication of the rank deficiency in the estimation of non-circular signals. In this paper, the resolution probability and RMSE are considered. In our simulations, all signals have the same power, and the signal-to-noise ratio (SNR) is defined as

(74) $$SNR=10\mathrm{lg}\left(\frac{\sum_{i=1}^{M}s_{i}^{2}}{\sum_{j=1}^{N^{\prime }}n_{j}^{2}}\right)$$

where $s_{i}^{2}$ is the power of the *i*-th signal and $n_{j}^{2}$ is the noise power of the *j*-th sensor.

### 4.1. Comparison of Resolution Probability

**Figure 4:**
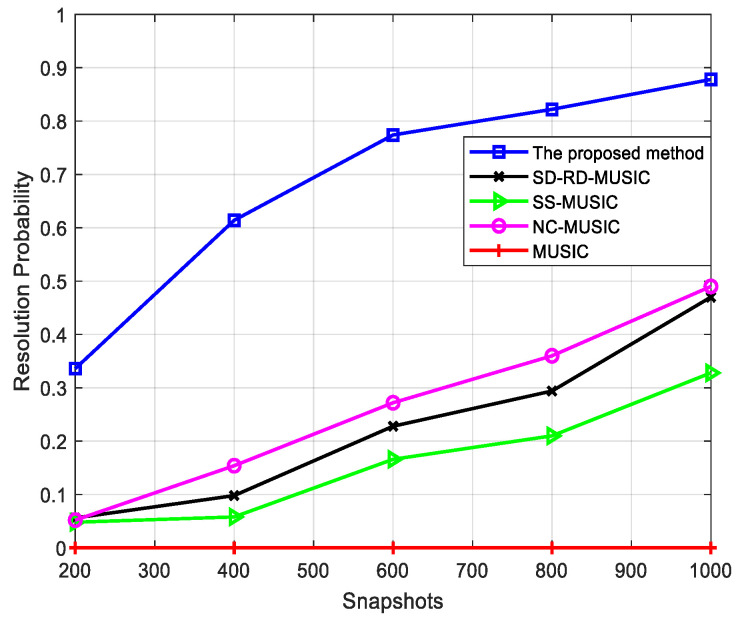
Comparison of resolution probability versus snapshots for two mixed signals, where SNR = −14 dB and angle interval = 2°.

The resolution probability when circular and non-circular signals have similar DOAs is analyzed first. In this simulation, when the angle error is less than $\frac{\left|\theta_{n}-\theta_{c}\right|}{2}$, the estimation is considered to be successful [17], where $\theta_{c}$ denotes the DOA of the circular signal and $\theta_{n}$ denotes the DOA of the non-circular one. Figure 4 shows the resolution probability versus the number of snapshots when SNR is −14 dB, where $\theta_{c}=20^{\circ }$, $\theta_{n}=22^{\circ }$, the non-circular signal phase $\varphi {=5}^{\circ }$, and $N_{1}=N_{2}=4$. From simulation results of 500 Monte Carlo experiments, the proposed method has the highest resolution probability and obvious advantages compared with other methods.

**Figure 5:**
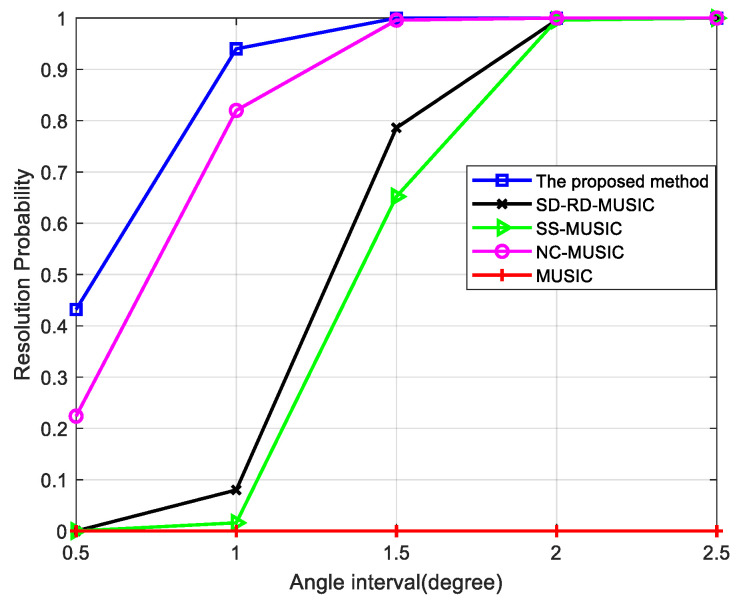
Comparison of resolution probability versus angle interval for two mixed signals, where SNR = 0 dB and snapshots = 200.

When the SNR is 0 dB and the number of snapshots is 200, the resolution probability of different angle intervals is simulated. As shown in Figure 5, when the angle interval is $0.5^{\circ }$, the resolution probability of the proposed method is more than 0.4, which is the highest among all methods. With the increase of the angle interval, the proposed method maintains the highest resolution probability.

From the above simulations, the proposed method has the highest resolution probability, and the advantage is more obvious with few snapshots and low SNR. Unlike other methods where all signals are estimated simultaneously, the separate estimation of different types of signals in our method increases the dimensions of the noise subspace in calculating the DOAs of non-circular signals and the two types of signals with similar DOAs can be distinguished. Our method achieves the highest resolution probability through the two-stage estimation.

**Figure 6:**
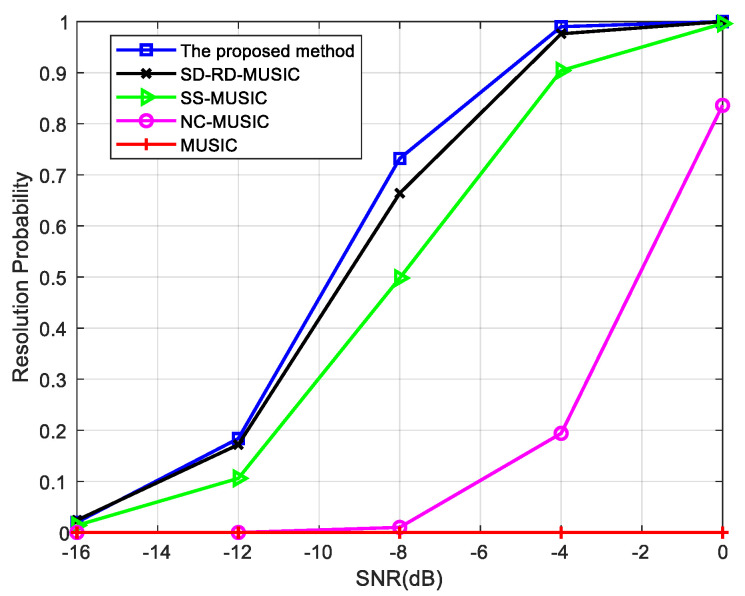
Comparison of resolution probability versus SNR for two non-circular signals, where snapshots = 200, angle interval = 2°.

Next, the resolution probability is simulated when the same types of signals impinge on the array. The resolution probabilities of different methods by estimating the two non-circular signals simultaneously are compared in Figure 6, where $\theta_{n}=\{20^{\circ },\;22^{\circ }\}$ and $\varphi {=\{0}^{\circ },{\;5}^{\circ }\}$.

Figure 6 also shows that our method has the best performance, and the highest resolution probability of the proposed method reflects the advantages of searching for the maximum condition number method.

### 4.2. Comparison of RMSE

In this section, the *RMSEs* of different methods are compared to analyze the accuracy of the proposed algorithm.

(75) $$RMSE=\sqrt{\frac{1}{VM}\left(\sum_{m=1}^{M}\sum_{v=1}^{V}{\left({\hat{\theta }}_{m,v}-\theta_{m}\right)}^{2}\right)}$$

where *V* is the simulation times, *M* is the number of signals, ${\hat{\theta }}_{m,v}$ is the estimated value of the *m*-th signal angle in the *v*-th experiment, and $\theta_{m}$ is the theoretical value of the *m*-th angle.

The performance of RMSE versus SNR and the snapshot number is simulated through 500 Monte Carlo experiments, where the DOAs and rotation phases of sources are shown in Table 1 and $N_{1}=N_{2}=4$.

**Figure 7:**
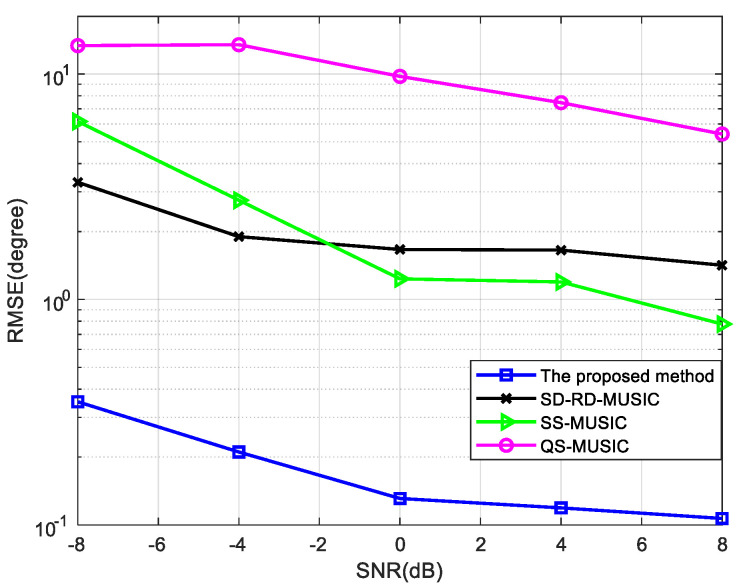
Comparison of RMSE versus SNR for four mixed signals, where snapshots = 1000.

**Figure 8:**
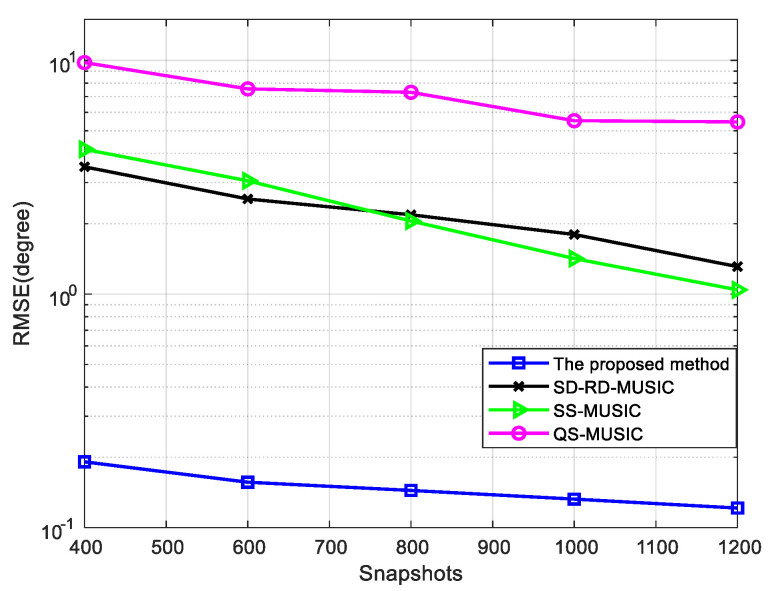
Comparison of RMSE versus snapshots for four mixed signals, where SNR = 0 dB.

When the number of snapshots is 1000, the RMSE is shown in Figure 7. When the SNR is 0 dB, Figure 8 shows the curve of RMSE versus the number of snapshots. It can be indicated that the proposed method has the best performance.

Unlike the SS-MUSIC method in [14], where only the cDCA is used, the cDCA and cSCA are exploited simultaneously in our method. Furthermore, the circular signals are eliminated before calculating the DOAs of non-circular ones in our method and the RMSE is smaller than estimating all signals together in [14,17]. The small number of snapshots in [18] (QS-MUSIC) leads to poor accuracy. The RMSE of the methods in [2,5] are more than $10^{\circ }$. Thus, the proposed method has the best performance.

When only a circular and a non-circular signal have similar DOAs, the proposed method has the minimum RMSE. If there are many signals in different types with similar or the same DOAs, the advantages of our method will be more obvious.

**Figure 9:**
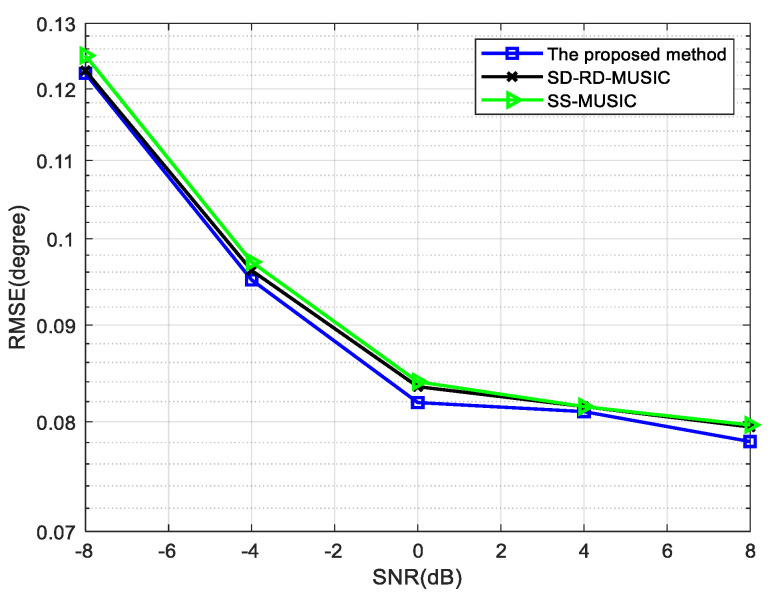
Comparison of RMSE versus SNR for four mixed signals, where snapshots = 800.

**Figure 10:**
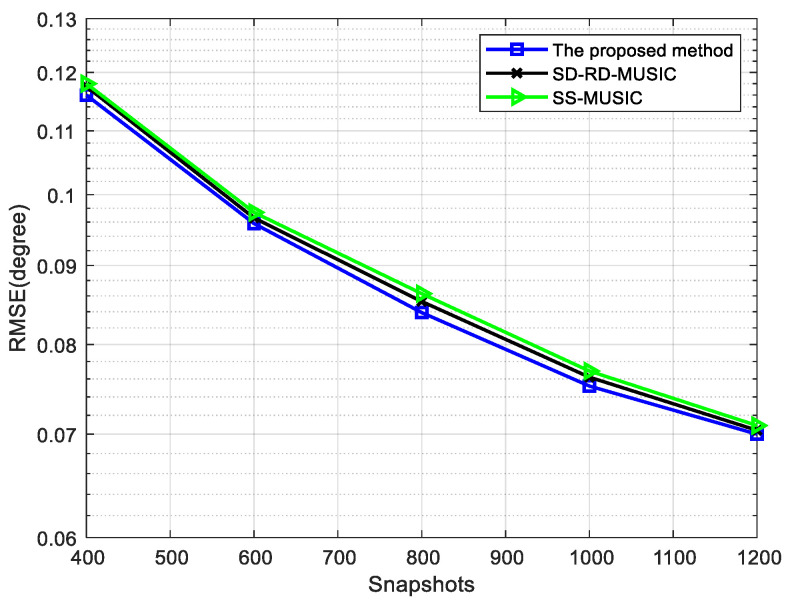
Comparison of RMSE versus snapshots for four mixed signals, where SNR = 0 dB.

Next, the RMSE is verified in Figure 9 and Figure 10 when the DOAs of circular signals and non-circular signals are distributed uniformly. In this simulation, the source information is shown in Table 2 and $N_{1}=N_{2}=4$.

Figure 9 shows the performance of RMSE versus SNR for different methods when the number of snapshots is 800. Figure 10 shows the RMSE when the number of snapshots is changed from 400 to 1200 under the condition of SNR = 0 dB and the other conditions remain unchanged. The RMSE of three methods decreases with the increase of the number of snapshots and our method has the best performance. The dimensions of the noise subspace reach the maximum for non-circular signals in our method, which can improve the accuracy of estimation.

When the angle interval of different types of signals becomes larger, the RMSEs of the different methods are close, because the interference between adjacent impinging signals has been reduced. Of course, in this case, the one-stage angle search without eliminating circular signals can also be exploited in our method with low computational burden.

**Figure 11:**
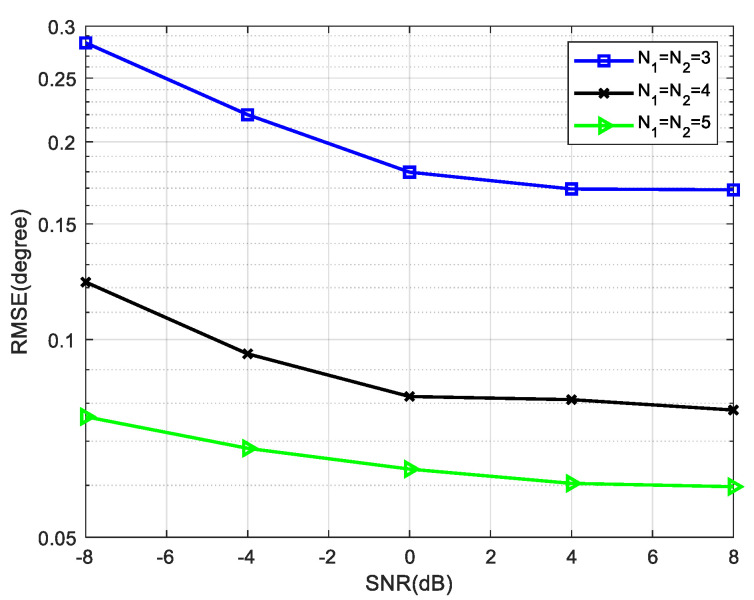
Comparison of RMSE versus SNR for four mixed signals, where snapshots = 800.

The relationship between the RMSE and the number of array elements is simulated, and three different cases are compared. The sources information is shown in Table 2. Figure 11 shows the change of RMSE with SNR, and the snapshot number is fixed at 800. It can be seen from the simulation results that when SNR is fixed, the RMSE decreases with the increase of array elements.

**Figure 12:**
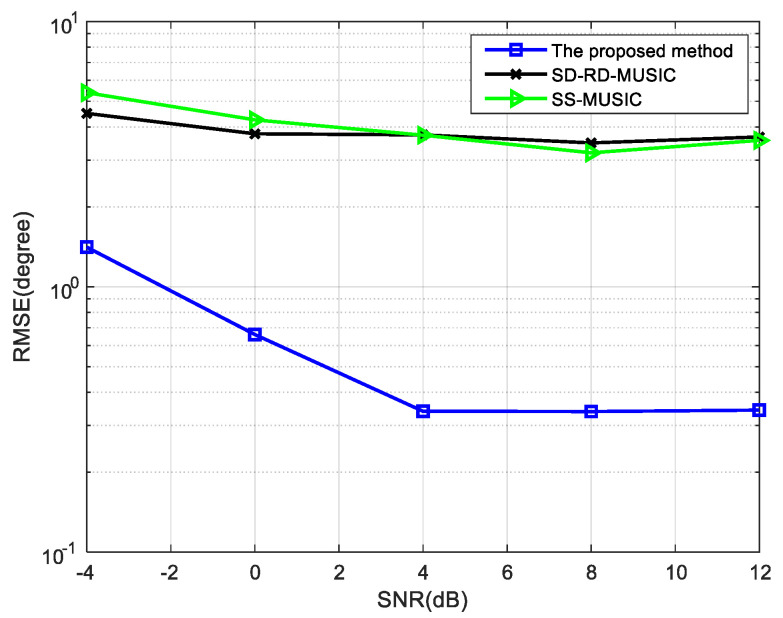
Comparison of RMSE versus SNR for nine mixed signals, where snapshots = 1000.

To test the performance of the method when the number of signals is more than the actual number of elements of the nested array, further simulations are carried out. When $\theta_{c}{=\{28}^{\circ }\}$ and $\theta_{n}=\{-40^{\circ },-30^{\circ },-20^{\circ },-10^{\circ }{,\;0}^{\circ }{,\;10}^{\circ },30^{\circ },40^{\circ }\}$, Figure 12 shows the RMSE of the three methods and $N_{1}=N_{2}=4$.

The RMSE of the proposed method is always the smallest and can achieve better accuracy when the number of snapshots and SNR are increased, as all signals can be well distinguished by the two-stage estimation method and the dimensions of the noise subspace are increased by eliminating the circular signals first. In [14,17], the large error makes it unable to distinguish multiple spectral peaks when there are many signals. Even if SNR or the number of snapshots is increased, they cannot obtain the correct estimation of DOAs.

**Figure 13:**
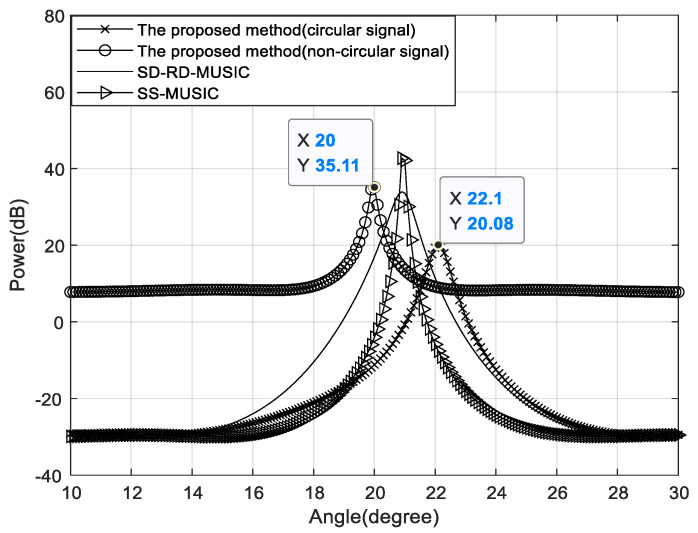
The spatial spectrum for two signals with SNR = −12 dB and 500 snapshots.

We also analyze the spatial spectrum of the proposed method when a circular signal and a non-circular signal have similar DOAs in Figure 13, where $\theta_{c}=22^{\circ }$ and $\theta_{n}=20^{\circ }$. The SNR is −12 dB, the number of snapshots is 500 and $N_{1}=N_{2}=4$.

The DOA estimation results of circular and non-circular signals in the proposed method are marked on Figure 13. Unlike other related methods, our method can always distinguish between two spectral peaks with similar DOAs and has high accuracy.

The above methods are comprehensively compared in Table 3 and the superiority of the proposed method is illustrated.

**Figure 14:**
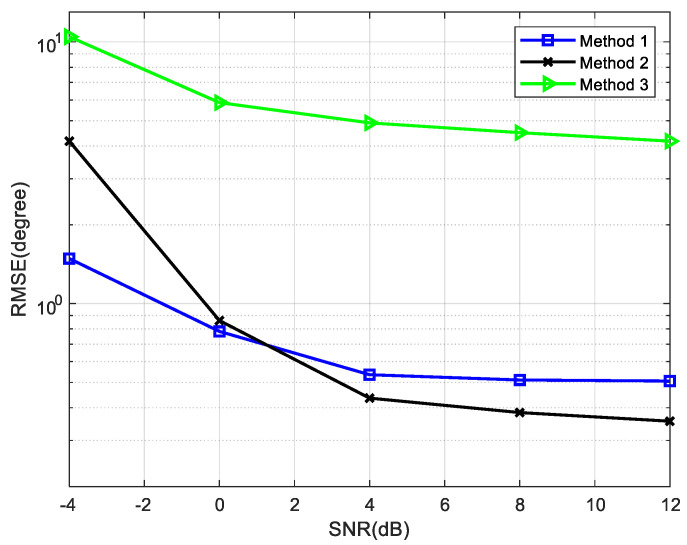
Performance comparison of different methods.

The rank deficiency is exploited in calculating the DOAs of non-circular signals; therefore, some different search methods can be evolved. Three representative methods are simulated in Figure 14. Circular and non-circular signals are estimated simultaneously.

Method 1: Find the peak value of the ratio of the maximum singular value to the minimum singular value (the condition number).

Method 2: Find the peak value of the ratio of the sub small singular value to the minimum singular value.

Method 3: Find the peak value of the reciprocal of the minimum singular value.

The variation of RMSE with SNR is analyzed in Figure 14, where the number of snapshots is 800, $N_{1}=N_{2}=4$ and the source information is shown in Table 4. When different non-circular signals have similar DOAs, method 1 and method 2 both have high accuracy. Thus, the method of searching for the maximum condition number is selected to calculate the DOAs in this paper.

## 5. Conclusions

In this paper, an algorithm is proposed to locate circular and non-circular signals separately with the nested array by exploiting different steering vectors of two types of signals. Only the DOAs of circular signals are estimated in the first stage after the construction of equivalent received signals on the cDCA and cSCA. After eliminating the information of circular signals, the dimensions of noise subspace are increased in estimating DOAs of non-circular signals in the second stage and signals with two types are distinguished. By exploiting the rank deficiency, three ways are proposed to estimate the DOAs of non-circular signals and the method of searching for the maximum condition number is chosen, where the computational burden is significantly reduced by converting 2-D search to 1-D search. Compared with some other methods such as SD-RD-MUSIC and SS-MUSIC, the proposed method has the highest resolution probability and the best estimation accuracy because of the two-stage estimation, especially when two types of sources have similar or the same DOAs. Numerical simulation results demonstrate that the proposed method is more effective and has better performance than other ones.

## Figures and Tables

**Table 1:** Information of the sources.

Source Types	DOA/(Degree)	Rotation Phase/(Degree)
circular	28, 40	0, 0
non-circular	10, 30	0, 5

**Table 2:** Information of the sources.

Source Types	DOA/(Degree)	Rotation Phase/(Degree)
circular	20, 40	0, 0
non-circular	10, 30	0, 5

**Table 3:** Comparison of different methods.

Method	The Way of Estimating Two Types of Signals	Whether the Types of Signals Can Be Distinguished	Whether the Rotation Phase Can Be Obtained
The proposed	separately	yes	yes
SD-RD-MUSIC	simultaneously	no	yes
SS-MUSIC	simultaneously	no	no
QS-MUSIC	simultaneously	no	no

**Table 4:** Information of the sources.

Source Types	DOA/(Degree)	Rotation Phase/(Degree)
circular	20, 40	0, 0
non-circular	28, 30	0, 5

## Data Availability

All data are presented in this article in the form of figures and tables.

## Appendix A

The projection operator can be constructed as [25]

(A1) $${Ε}_{A_{SC}(\theta )A_{SN}(\theta )}=A_{SC}(\theta ){\left(A_{SC}^{H}(\theta )P_{SN}^{⊥}A_{SC}(\theta )\right)}^{-1}A_{SC}^{H}(\theta )P_{SN}^{⊥}$$

where

(A2) $$P_{SN}^{⊥}=I-A_{SN}(\theta )A_{SN}^{†}(\theta )$$

(A3) $$\begin{array}{ll} Y_{u} & =E_{A_{SC}(\theta )A_{SN}(\theta )}Y\\ & =A_{SC}(\theta ){\left(A_{SC}^{H}(\theta )P_{SN}^{⊥}A_{SC}(\theta )\right)}^{-1}A_{SC}^{H}(\theta )P_{SN}^{⊥}Y\\ & =A_{SC}(\theta )S_{DC}+E_{A_{SC}(\theta )A_{SN}(\theta )}N_{q} \end{array}$$

Then

(A4) $$Y-Y_{u}=(I-E_{A_{SC}(\theta )A_{SN}(\theta )})Y=A_{SN}(\theta )S_{DN}+(I-E_{A_{SC}(\theta )A_{SN}(\theta )})N_{q}$$

$S_{DC}$ is the circular signals part and $S_{DN}$ is the non-circular signals part.

It can be seen from (A1) that to calculate the oblique projection operator ${Ε}_{A_{SC}(\theta )A_{SN}(\theta )}$. It is necessary to know the DOAs of all sources, but they cannot be obtained in the actual environment, so the following formula is used as the approximate expression of ${Ε}_{A_{SC}(\theta )A_{SN}(\theta )}$.

(A5) $${Ε}_{A_{SC}(\theta )A_{SN}}=A_{SC}(\theta ){\left(A_{SC}^{H}(\theta )R_{\chi }^{†}A_{SC}(\theta )\right)}^{-1}A_{SC}^{H}(\theta )R_{\chi }^{†}$$

Then the covariance matrix can be expressed as

(A6) $$\begin{array}{l} R_{\nu }=\frac{1}{R_{1}+1}(Y-Y_{u}){\left(Y-Y_{u}\right)}^{H}\\ =\frac{1}{R_{1}+1}[(I-E_{A_{SC}(\theta )A_{SN}})Y]{\left[(I-E_{A_{SC}(\theta )A_{SN}})Y\right]}^{H}\\ =(I-E_{A_{SC}(\theta )A_{SN}})(\frac{1}{R_{1}+1}YY^{H}){\left(I-E_{A_{SC}(\theta )A_{SN}}\right)}^{H}\\ =(I-E_{A_{SC}(\theta )A_{SN}})R_{\chi }{\left(I-E_{A_{SC}(\theta )A_{SN}}\right)}^{H} \end{array}$$

## References

[1] Magiera J. (2021). Detection and direction-of-arrival estimation of weak spread spectrum signals received with antenna array. Electronics.

[2] Schmidt R.O. (1986). Multiple emitter location and signal parameter estimation. IEEE Trans. Antennas Propag..

[3] Wang R., Wang Y., Li Y.P., Cao W.M., Yan Y. (2021). Geometric algebra-based ESPRIT algorithm for DOA estimation. Sensors.

[4] Tan Y.W., Wang K., Wang L.L., Wen H. (2021). Efficient FFT based multi source DOA estimation for ULA. Signal Process..

[5] Abeida H., Delmas J.P. (2006). MUSIC-like estimation of direction of arrival for noncircular sources. IEEE Trans. Signal Process..

[6] Guo M.R., Zhang Y.D., Chen T. (2018). DOA estimation using compressed sparse array. IEEE Trans. Signal Process..

[7] Chung H., Seo H., Joo J., Lee D., Kim S. (2021). Off-grid DOA estimation via two-stage cascaded neural network. Sensors.

[8] Al Mahmud T.H., Ye Z.F., Shabir K., Zheng R., Islam M.S. (2018). Off-grid DOA estimation aiding virtual extension of coprime arrays exploiting fourth order difference co-array with interpolation. IEEE Access.

[9] Chen T., Shi L., Guo L.M. (2019). Sparse DOA estimation algorithm based on fourth-order cumulants vector exploiting restricted non-uniform linear array. IEEE Access.

[10] Zhao P.J., Hu J.B., Qu Z.Y. (2019). Enhanced nested array configuration with hole-free co-array and increasing degrees of freedom for DOA estimation. IEEE Commun. Lett..

[11] Han P., Xu H.Y., Ba B. (2020). Direction-of-arrival estimation in a mixture of multiple circular and non-circular signals using nested array. IEEE Access.

[12] Zheng W., Zhang X.F., Li J.F., Shi J.P. (2021). Extensions of co-prime array for improved DOA estimation with hole filling strategy. IEEE Sens. J..

[13] Li J., Zhao J., Ding Y.H., Li Y.F., Chen F.J. (2021). An improved co-prime parallel array with conjugate augmentation for 2-D DOA estimation. IEEE Sens. J..

[14] Pal P., Vaidyanathan P.P. (2010). Nested arrays: A novel approach to array processing with enhanced degrees of freedom. IEEE Trans. Signal Process..

[15] Gong P., Chen X.X. (2022). Computationally efficient direction-of-arrival estimation algorithms for a cubic coprime array. Sensors.

[16] Wang Y.F., Wu W., Zhang X.F., Zheng W. (2020). Transformed nested array designed for DOA estimation of non-circular signals: Reduced sum-difference co-array redundancy perspective. IEEE Commun. Lett..

[17] Wang Y.F., Shen J.Q., Zhang X.F., He Y., Dai X.R. (2020). Non-circular signals for nested array: Sum-difference co-array and direction of arrival estimation algorithm. IET Radar Sonar Nav..

[18] Huang H.P., Liao B., Wang X.Y., Guo X.S., Huang J.J. (2018). A new nested array configuration with increased degrees of freedom. IEEE Access.

[19] Zhang X.F., Wang Y.F., Zheng W. Direction of arrival estimation of non-circular signals using modified nested array. Proceedings of the 2020 IEEE 11th Sensor Array and Multichannel Signal Processing Workshop (SAM).

[20] Xie Y., Huang M., Zhang Y.Y., Duan T., Wang C.Y. (2021). Two-stage fast DOA estimation based on directional antennas in conformal uniform circular array. Sensors.

[21] Zhao Y.Q., Shen F., Xu G.H., Wang G.C. (2021). A spatial-temporal approach based on antenna array for GNSS anti-spoofing. Sensors.

[22] Liu J., Huang W.C., Wang L., Shi Z.P. Wideband spectrum sensing based on nested sampling for noncircular signals. Proceedings of the IEEE 20th International Conference on Communication Technology (ICCT).

[23] Schreier P.J., ScharfL L. (2010). Statistical Signal Processing of Complex-Value Data: The Theory of Improper And Non-Circular Signals.

[24] Zheng Z., Fu M.C., Jiang D., Wang W.Q., Zhang S. (2018). Localization of mixed far-field and near-field sources via cumulant matrix reconstruction. IEEE Sens. J..

[25] Behrens R.T., Scharf L.L. (1994). Signal processing applications of oblique projection operators. IEEE Trans. Signal Process..

